# Modulation of Bacterial Multidrug Resistance Efflux Pumps of the Major Facilitator Superfamily

**DOI:** 10.1155/2013/204141

**Published:** 2013-12-05

**Authors:** Sanath Kumar, Mun Mun Mukherjee, Manuel F. Varela

**Affiliations:** ^1^QC Laboratory, Harvest and Post Harvest Technology Division, Central Institute of Fisheries Education (CIFE), Seven Bungalows, Versova, Andheri (W), Mumbai 400061, India; ^2^Biology Department, Eastern New Mexico University, Portales, NM 88130, USA

## Abstract

Bacterial infections pose a serious public health concern, especially when an infectious disease has a multidrug resistant causative agent. Such multidrug resistant bacteria can compromise the clinical utility of major chemotherapeutic antimicrobial agents. Drug and multidrug resistant bacteria harbor several distinct molecular mechanisms for resistance. Bacterial antimicrobial agent efflux pumps represent a major mechanism of clinical resistance. The major facilitator superfamily (MFS) is one of the largest groups of solute transporters to date and includes a significant number of bacterial drug and multidrug efflux pumps. We review recent work on the modulation of multidrug efflux pumps, paying special attention to those transporters belonging primarily to the MFS.

## 1. Introduction

Drug and multidrug resistant bacterial pathogens that are causative agents of infectious disease constitute a serious public health concern. Bacterial multidrug efflux pump systems of the major facilitator superfamily (MFS) and resistance-nodulation-cell division (RND) superfamily represent common mechanisms for bacterial resistance to antimicrobial agents. As such these bacterial transporters make suitable targets for modulation in order to restore the clinical efficacy of relevant chemotherapeutic antibacterial agents. Here, we briefly review the drug transporter systems of the MFS (and to a lesser extent the RND superfamily) and discuss their modulation via regulation of expression and efflux pump transport inhibition.

## 2. Bacteria and Pathogenesis

Bacteria are unicellular, microscopic living organisms that are rod shaped, ball shaped, or spiral shaped when observed under the microscope. Most bacteria are not harmful; rather, they aid in food preparation and digestion, compete with pathogens, provide vitamins to the body, are useful for basic and applied research purposes, and are important in biotechnology. However, less than one percent of the bacteria of different types are responsible for causing bacterial infections. Bacterial cells are capable of quickly reproducing and releasing chemicals and toxins; pathogenic bacteria can cause damage to cells and tissues in the body and cause clinical disease. Some of the common diseases and infections caused by pathogenic strains of bacteria include food poisoning caused by *Escherichia coli* and *Salmonella *[1–6], gastritis and ulcers caused by *Helicobacter pylori *[7], the sexually transmitted disease gonorrhea caused by *Neisseria gonorrhoeae* [8], meningitis caused by *N. meningitides* [9], skin infections like boils, cellulitis, abscesses, wound infections, toxic shock syndrome, pneumonia, and food poisoning caused by *Staphylococcus aureus *[10–13], and pneumonia, meningitis, otitis, and strep throat caused by streptococcal bacteria [14–16]. Thus, it is important to investigate bacterial mechanisms that confer pathogenesis in order to reduce the conditions that foster their emergence and movement through populations.

## 3. Bacterial Resistance Mechanisms to Antimicrobial Agents

**Figure 1:**
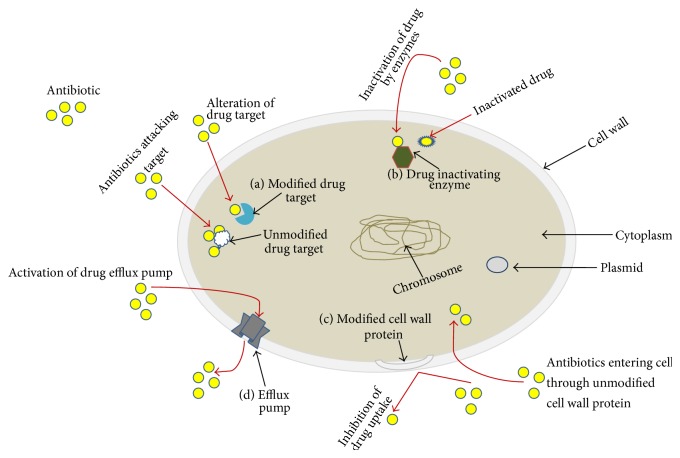
Mechanisms representing antibacterial resistance. A generic bacterium is depicted in which various mechanisms for resistance to antimicrobial agents are indicated. (a) Drug target modification, (b) drug inactivation by enzymes, (c) reduced drug permeability by membrane modification, and (d) active efflux of drugs from the bacterial cell. Yellow circles indicate antimicrobial agent molecules; red arrows indicate movement of molecules, and black arrows are pointing to intra- and extracellular structures.

Antimicrobial chemotherapy is frequently indicated for infections caused by the bacteria mentioned above and others [17]. Bacterial resistance, however, to antimicrobial agents has emerged in many of these pathogens, often confounding treatment efforts [18]. Bacterial pathogens that are resistant to a single drug are also quite frequently resistant to multiple antimicrobial agents and are considered potentially untreatable “superbugs” [19, 20]. Even though some efforts are underway to overcome this problem by developing new lines of antibiotics with novel mechanisms and newly improved activities, bacteria are nonetheless quickly acquiring resistance determinants and are prevailing as multidrug resistant pathogens [21–25]. In the competition between bacteria and antimicrobial agents, pathogenic bacteria are thought to have an upper hand by transferring drug resistance genetic determinants between distinct bacterial species and acquiring resistant phenotypes against most antimicrobial agents [26–29]. The spread of antibiotic resistance in the last decade has been a major challenge when dealing with human health concerns [30]. Releasing antibiotics into the environment is also a major cause in the development and emergence of bacterial antibiotic resistances [31–36]. Inappropriate use and misuse of antimicrobials can foster conditions in which less susceptible bacterial variants survive, become adapted to low drug concentrations, and eventually develop resistance [37, 38]. Interestingly, a bacterial strain that is selected as a single-drug resistant variant by exposure to a single drug is frequently multidrug resistant to antimicrobials that are structurally distinct from the original selective drug [39–42]. General mechanisms which are responsible for bacterial resistances to antimicrobial agents are shown in Figure 1 and include (a) alteration of the sites where the drugs are targeted, (b) enzymes that inactivate the antibiotics, (c) decreased membrane permeability, and (d) active efflux of antimicrobials. Bacterial resistance mechanisms such as these mentioned above provide investigators with good cellular targets for potential modulation. Studies of the modulatory effects on bacterial drug resistance mechanisms, especially those dealing with multidrug resistances, may lead to restoration of the efficacy of antimicrobial agents that have previously been less than efficacious in multidrug resistant pathogens.

## 4. Antimicrobial Efflux Pumps of Bacteria

**Figure 2:**
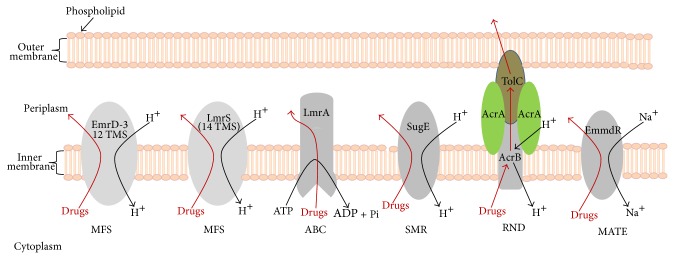
Antibacterial resistance by multidrug efflux pumps. Transporters of the MFS are capable of carrying solutes across the biological membrane, and the energy for solute translocation comes from the chemiosmotic gradient of cations [58–60]. EmrD-3 from *V. cholerae* [61] represents a MFS multidrug efflux pump (a drug/H^+^ antiporter) with 12 transmembrane domains, and LmrS from *S. aureus* [62] represents a MFS drug/H^+^ antiporter with 14 TMS. The transporters of the ABC superfamily can transport ions, small molecules, and macromolecules in and out of the cell using the hydrolysis of ATP [63, 64]. The SMR family members confer resistance to quaternary ammonium compounds as well as a variety of antibiotics and are represented by SugE [65, 66]. The RND superfamily of tripartite efflux pumps works by cation gradients and can be found in both Gram-positive and Gram-negative bacteria [67, 68]. The MATE superfamily of drug efflux pumps extrudes antibiotics out of the bacterial cell via cation gradients and is represented by EmmdR [69, 70]. Both outer and inner (cytoplasmic) membranes are shown for illustration purposes, as some of the transporters are found in Gram-positive bacteria which lack a second membrane. For clarity, the peptidoglycan is not shown.

Active efflux as a mechanism for bacterial resistance to inhibitory substances, such as toxic compounds and antibiotics, is mediated by integral membrane transporters, known simply as drug efflux pumps [43]. There are several main categories of active drug efflux pumps that transport drugs against their concentration gradients across the membrane; see Figure 2. The first category consists of pumps, called primary active transporters, which utilize the energy stored in ATP to catalyze transport of drug across the membrane by ATP hydrolysis [44]. The second category consists of pumps, called secondary active transporters, which are driven by the energy stored in ion gradients that are in turn generated by respiration, to catalyze the transport of drug across the membrane [45–48]. These primary and secondary active drug efflux pumps are largely responsible for conferring antibacterial resistances, and in many cases multiple drug resistances [49–51]. These efflux pumps are located on the cytoplasmic or plasma membranes of bacteria and prevent drug accumulation inside the bacterial cells, thereby conferring resistance [40]. A third category of drug pumps, called the phosphotransferase system (PTS), catalyzes the transport of drug with a concomitant phosphorylation of the drug, usually for cellular entry of the drug substrate [52, 53]. Bacterial genome sequencing projects facilitate the identification of the putative genes responsible for building antimicrobial resistance [54–56]. The genes responsible for building resistance are collectively called a “resistome” [57].

Based on the modes of energy, amino acid sequence similarities, predicted secondary protein structures, known 3D crystal protein structures, and phylogenetic relationships, bacterial drug efflux transporters are classified into five different major superfamilies and are shown in Figure 2: (i) the major facilitator superfamily (MFS) [58, 71]; (ii) the ATP-binding cassette (ABC) superfamily [72, 73]; (iii) the small multidrug resistance (SMR) superfamily [74]; (iv) the resistance-nodulation-cell division (RND) superfamily [75, 76]; and (v) the multidrug and toxic compound extrusion superfamily (MATE) of transporters [69].

## 5. The Major Facilitator Superfamily

The major facilitator superfamily (MFS) of transporters comprises uniporters, symporters, and antiporters and has been called the uniporter-symporter-antiporter (USA) family [77]; see Table 1. The MFS was discovered by Henderson and coworkers [78–80]. These investigators found the seemingly distinct transporters of diverse substrates shared similar deduced amino acid sequences, predicted secondary protein structures within the membrane, and evolutionary relatedness [46, 80–83]. Since their initial discovery, the MFS of transporters has become an important and intensive area of investigation [50, 58–60, 71, 84, 85]. Since many members of the MFS confer bacterial drug and multidrug resistance, these transporters collectively represent a good system for the study of modulation, both at the level of gene expression and of inhibition of drug transport across the membrane. Both of these avenues hold promise for eventually restoring the clinical efficacy of clinically important antimicrobial agents.

### 5.1. MFS Multidrug Efflux Pumps

The efflux proteins of the MFS (Table 1) belong to the antiporter group, which may be comprised of either monomeric (e.g., *qacA*/B, *mdfA*, and *emrD*-3) or multicomponent systems (e.g., EmrAB-TolC) [86]. The genes encoding these efflux pumps are largely chromosomal, but some others such as *qacA/B* are plasmid borne [121]. About half of the 39 putative drug efflux pumps in the *E. coli* genome are of the MFS-type, which is about 10% of all the proteins encoded in the whole genome of this organism [122, 123]. With the whole genomes of several bacteria available now in the public databases, homologues of known efflux pumps can be identified easily by BLAST searches. For example, homologues of EmrD-3, a *Vibrio cholerae* multidrug efflux pump [61] with >65% amino acid identity, could be found in whole genome sequences of a large number of Gram-negative bacteria belonging to *Vibrio*, *Shewanella*, aeromonad, enterobacteria, pseudomonad, *Moraxella,* and *Alcaligenes* groups. Similarly, a BLAST search using the multidrug efflux pump from a clinical isolate of *Staphylococcus aureus* (LmrS) [62] identified homologues of this protein in the whole genome sequences of many species of Gram-negative and -positive bacteria, especially in genomes of *Staphylococcus* and *Bacillus*. However, it is not known if these homologous proteins have similar substrate profiles, a feature highly unpredictable in the case of drug and multidrug efflux pumps.

#### 5.1.1. Regulation of Efflux Pump Expression

Though, by far, efflux pumps may not confer clinical levels of resistance to antimicrobials, their actions reduce the intracellular concentrations of antibiotics to sublethal levels leading to the development of specific modes of resistance *via* gene mutations or antibiotic degradation [124, 125]. However, exceptions to this general perception, especially in Gram-positive bacteria, have made efflux pumps clinically relevant and are the focus of intense research [124]. Such efflux pumps are either constitutively expressed or expressed at a higher level in clinical isolates or are induced to express at a higher level due to prolonged exposure to antimicrobial compounds [126]. Some of the efflux pump genes are under the tight control of regulators which control their expressions. The genes encoding efflux protein and the regulator of the efflux pump coexist and have overlapping promoters as seen in tetracycline efflux pumps [127]. When there is no antibiotic, the tetracycline repressor prevents the transcription of both efflux and regulatory gene by binding to the operator region. When present in the growth medium, tetracycline binds to the repressor protein preventing it from interacting with the operator thus allowing the transcription of *tetA* structural genes [127].

*The acr and mar Loci Gene Expression.* In some instances, global regulatory mechanisms control the expression of efflux pumps, and as a consequence of this, any single mutation in the regulator gene can lead to several efflux pumps being up- or downregulated in a single bacterium [128, 129]. In *E. coli,* for example, expression of some of the efflux pumps responsible for bile resistance is regulated by *acr* and *mar* loci [130]. Constitutive expression of *marA* or its orthologs *soxS* and *ramA* in some pathogenic bacteria such as *Salmonella* Typhimurium, *Klebsiella pneumoniae*, and *E*. *coli* could make these microorganisms resistant to organic solvents and multiple drugs [131, 132]. The role of *marA* and its orthologs has been confirmed by gene deletion experiments in which deletion mutants were found to be more virulent than the wild types [133]. Okusu et al. [134] discovered that the *marA*-mediated antibiotic resistance was in fact due to the increased expression of the *acrAB* efflux pump in *E. coli*. Following this, the roles of *marA*, *acrR,* and *ramA* in antibiotic resistance *via* the overexpression of efflux pumps have been reported from other Enterobacteriaceae [135, 136]. In clinical isolates of *E. coli,* a frame shift mutation in *marR* was responsible for the constitutive overexpression of *marA* and *acrAB* resulting in tigecycline resistance [137]. Deletion of AraC-like protein-encoding genes dramatically decreased intestinal colonization in a mouse model [138], while in the case of *S*. Typhimurium DT104, deletion of the gene encoding MarA resulted in the reduced survival in chicken spleen and caecum [139]. These data clearly suggest that multiple virulence genes and genes necessary for survival and colonization are regulated by the *araC* group of proteins. Likewise, deletion of *araC *orthologues in other pathogenic bacteria such as *P. aeruginosa* (ExsA), *V. cholerae* (ToxT), and *Yersinia pestis* (LcrF) also resulted in attenuation of these pathogens in mouse models [140–142]. The *araC* family of transcriptional regulators such as MarA can regulate, positively or negatively, the expression of several genes including virulence and MDR genes [143]. Due to their critical roles in the survival and virulence of pathogenic bacteria, the *araC* family or similar regulons make ideal targets for the inhibitors.

The fact that overexpression of efflux pumps is induced by the antibiotics during the course of treatment is a serious concern, since such bacteria may show antibiotic sensitivity in laboratory tests. The problem is confounded when efflux pumps are overexpressed by unrelated antibiotics and even antimicrobials like disinfectants and household chemicals [86, 125, 144]. A similar phenomenon has also been observed in gastrointestinal *E. coli* [145]. The overexpression of TolC has been found to be responsible for clinical *Shigella* developing fluoroquinolone resistance [146]. The decreased permeability to antibiotics *via* decreased porin expression has been associated with overexpression of AcrAB [136]. On the other hand, mutations in regulator genes may lead to unregulated expression of the efflux pump conferring constitutive multidrug resistance to the bacterium [147]. Thus, it is important to understand the mechanisms of efflux pump regulation, specifically during antimicrobial treatment. The overexpression of efflux pumps in some clinical strains is responsible for antibiotic resistance, and this mechanism has not been understood well [148]. In a clinical isolate of *K. pneumoniae*, the overexpression of KmrA and KdeA confers elevated tolerance to quinolones [149, 150].

*Bmr and blt Efflux Pumps of B. subtilis and Regulation of Expression*. It is intriguing that the bacteria have distinct regulatory mechanisms for homologous efflux pumps, and presumably the chemical and biological inducers of these regulators are also different as seen in the case of two homologous efflux pumps *bmr* and *blt* of *B. subtilis*. *bmr* is constitutively expressed, while *blt* is not expressed under normal growth conditions [151]. The expression of *bmr* is under the control of a regulatory gene *bmrR*, the protein product of which binds to the promoter upstream of *bmr* gene. The binding of *bmrR* is stronger in the presence of compounds such as Rhodamine 6G resulting in higher levels of expression of *bmr* [152]. A second regulator, BltR, which has no homology with *bmrR*, regulates the expression of *blt *[151].

*QacA of S. aureus and Regulation of Expression.* The role of multiple regulators on the expression of efflux pumps is well elucidated in *S. aureus* and has been recently and extensively reviewed by Schindler et al. [153]. QacA/B efflux pumps are some of the earliest discovered efflux pumps of the MFS family from *S. aureus* that confer resistance to biocides such as quaternary ammonium compounds [121, 154]. This efflux pump has been subjected to intense studies of its transmembrane structure, substrate binding domains, and amino acid residues critical for substrate binding and substrate efflux [50, 155–159]. The expression of *qacA* is controlled by a repressor protein QacR which is induced by structurally dissimilar compounds [160].

*NorA of S. aureus and Regulation of Expression. *The other important efflux pump of *S. aureus*, NorA, was initially discovered as a fluoroquinolone-specific pump [161] and later was found to transport several nonquinolone compounds [162]. Several other efflux pumps that are homologous to *norA* such as *norB* and *norC* have been discovered in *S. aureus*, and all these are negatively regulated by MgrA [110, 163]. The overexpression of *norA* in clinical isolates has been observed, and this is due to a mutation in the *norA* promoter that resulted in the inability of the regulator protein to bind to the promoter [164]. A two-component regulator ArlSR also has a role in *norA* expression, since its deletion from *S. aureus* resulted in constitutive expression of *norA* [165]. NorB is negatively regulated by MgrA but positively by NorG [166], though the deletion of *norG* did not change the fluoroquinolone resistance of *S. aureus* [166]. Though NorG binds to the promoters of *norA*, *norB*, *norC,* and *abcA* (a transporter of the ABC-family), its regulatory effect is more pronounced on NorB, since its overexpression resulted in a 3-fold increase in *norB* transcripts and a 4-fold increase in quinolone resistance [166]. This study showed that multiple regulators occurring in a single bacterium can have completely different regulatory roles on efflux pumps.

The development of resistance can occur when a bacterium is constantly exposed to an antibacterial agent. *S. aureus* exposed to increasing concentrations of ethidium bromide developed higher levels of resistance to fluoroquinolones and biocides compared to the parent strain, and this increased resistance was due to a several-fold increase in the expression of the *norA* efflux gene, which in turn was due to a 70 bp deletion in the *norA* promoter region [167].

## 6. Modulation of Efflux Pump Activity

Several studies have demonstrated the development of antibiotic resistance in pathogenic bacteria during the course of antibiotic treatment which involved efflux pumps [20, 25, 40, 132, 168]. Therefore, by hypothesis, the antibiotic therapy can be made effective if (i) efflux pumps are inhibited, (ii) the expression of efflux pumps is downregulated, or (iii) the antibiotics are redesigned, so that they are no longer suitable efflux substrates, and thus their clinical efficacy is restored [169].

One of the rational approaches towards confronting efflux of clinically relevant antibiotics is to discover or design potent efflux pump inhibitors. In line with the enzyme-substrate-competitive inhibitor concept, it may be hypothesized that if efflux pumps have natural inhibitors, they may also have artificial inhibitors. A number of known compounds have been identified as inhibitors of efflux pumps in addition to novel natural and synthetic products being reported as efflux pump inhibitors [170]. Some efflux pumps are essential for survival, biofilm formation, host colonization, and virulence, and hence their inhibition potentially affects bacterial pathogenesis [124]. To achieve this, critical information on molecular interactions between the efflux pumps and their drug targets, stoichiometry of the drug/proton antiport process, and the regulation of efflux gene expression itself are needed.

## 7. Inhibition of RND Bacterial MDR Efflux Pumps

A brief overview of the scientific literature suggests that new and novel efflux pumps and their preferred antimicrobial substrates are being reported regularly from pathogenic and nonpathogenic bacteria. However, studies to understand the molecular basis of their drug preference, 3-dimensional structures of the efflux pumps, and ways of overcoming them to make antimicrobial therapy more effective are not forthcoming in at the same pace. Despite the lack of physical data on 3-dimensional structures of efflux pumps, bioinformatics tools have helped to understand the efflux pump/drug or efflux pump/proton interactions during active transport to a greater extent. However, this approach also suffers from serious drawbacks when an efflux protein in question does not have close structural homology with proteins whose crystal structures have been determined [171, 172]. With multidrug resistance efflux pumps, determining the crystal structure for multiple antibiotics is a difficult task. Recent elucidation of crystal structures of some important efflux pumps have helped to understand the structure-function relationships in these pumps. The crystal structure of AcrB with bound minocycline and doxorubicin has been described [173, 174]. AcrB is a MDR efflux pump with multiple important substrates apart from those used for crystal structure derivation. Using docking tools, the interaction of the AcrB drug-binding pocket with several antibiotics has been studied, and this is an example of how bioinformatics tools can help understanding the efflux pump-drug interactions and the testing of potential efflux pump inhibitors (EPIs) [175]. This *in silico* study showed different binding pockets for different antimicrobials within the main protein domain [171]. This finding has far reaching implications in the efficacy of competitive EPIs, and due to differences in binding pockets for two different antibiotics of the same efflux pump, a competitive inhibitor may not be able to block the efflux of both antibiotics with the same efficiency [31, 176]. However, it must also be noted that the docking experiments and *in vitro* observations on the substrate specificity of efflux pumps may not always correlate as observed in the case of AcrAB-TolC and MexAB-OprM systems, in which the observed antibiotic specificity did not correlate well with the docking studies [177, 178], and such discrepancies can occur due to unique conformational changes in the efflux proteins upon drug binding which are not contemplated by the docking tools [178]. In Gram-negative bacteria, phenyl-arginine-*β*-naphthylamide (Pa*β*N) has been demonstrated to be a potent EPI and could diminish the norfloxacin resistance activities conferred by Mex efflux systems of *Pseudomonas aeruginosa* [179], the AcrAB efflux system of the Enterobacteriaceae family [176], and the erythromycin efflux system of *Campylobacter jejuni* [180].

The ability of a majority of antimicrobial efflux pumps to bind and transport a range of structurally different substrates offers both advantages and disadvantages. From a favorable perspective, there is a greater scope to screen structurally dissimilar compounds as inhibitors of efflux pumps. On the other hand, it is difficult to determine a single structural conformation responsible for drug efflux and to identify specific residues as critical for the transport of a range of substrates [178]. Nevertheless, the possibility remains of using some compounds as efflux pump inhibitors along with the antibiotics, so that the extrusion of the antibiotics does not take place, and thus sufficient intracellular concentration can kill the bacteria. This idea has gathered interest primarily because by doing so successfully, the antibiotics that are otherwise dismissed as ineffective can now be used again clinically [101, 176]. Quinolone derivatives used as competitive inhibitors of the AcrB efflux pump showed varying effects across different Enterobacteriaceae. Also, the effectiveness of an EPI will be different when being used with different antibiotics [181], and this may also depend on the level of expression of efflux pumps as well as the relative affinity of the antibiotic binding site for different antibiotics. Recently, the antimicrobial activity of tetracycline was enhanced by the addition of silver to bacterial cells that had been previously resistant to this antimicrobial agent, although the specific drug efflux pump system responsible was not definitively identified in this study [182].

## 8. Inhibition of MFS Bacterial MDR Efflux Pumps

Bacterial drug and multidrug efflux pumps of the MFS are common amongst clinically important pathogens [50, 51, 59, 60, 71, 183]. Multidrug resistant bacterial pathogens compromise the clinical utility of antimicrobial agents during treatment of their infectious disease [20]. Modulation of bacterial multidrug efflux pumps of the MFS would be, therefore, of tremendous importance in order to eventually restore the clinical utility of antimicrobial agents [170, 184].

### 8.1. CCCP and MFS MDR Pumps

One of the straightforward approaches to inhibiting efflux pumps is to prevent their energization by protons, such as in the case of drug/H^+^ antiporters. Since these efflux pumps are potentiated by protons, compounds which have proton scavenging activities can block the activity of these efflux pumps. A well known example of an inhibitor that uses this blocking of energization (energy uncoupler) as a mechanism is carbonyl cyanide *m*-chlorophenylhydrazone (CCCP), which is a proton-conducting uncoupler of the proton potential that dissipates the respiration-generated proton gradient and thus inhibits secondary active transporters [185]. CCCP and other proton conductors are frequently used during the initial physiological characterizations of newly discovered drug and multidrug efflux pumps to ascertain whether the new pumps are primary or secondary active transporters. Several natural compounds such as the plant alkaloid reserpine, kaempferol rhamnoside, and capsaicin inhibit NorA function [186, 187]. The mechanism of inhibition for reserpine and kaempferol rhamnoside involves direct binding and competitive inhibition of the efflux pump during drug/H^+^ antiport [188], but the mechanism for capsaicin-mediated inhibition is unclear. Several natural products have been shown to be potent EPIs and have been extensively reviewed elsewhere [86, 169, 189]. Some of the desirable properties of clinically useful EPIs are that they should be nontoxic to humans and non-human animals and should not lead to development of cross-resistance to other antibiotics [190], and therefore, careful selection and testing of EPIs are very critical.

### 8.2. Reserpine and MFS MDR Pumps

Reserpine has long been known to be a competitive inhibitor of both primary and secondary active transporter systems [191, 192]. One of the first transporters of the major facilitator superfamily to be analyzed with reserpine was a multidrug transporter from the Gram-positive bacterium *Bacillus subtilis*, *bmr* [193], which had previously been shown to transport ethidium bromide and confer resistance to structurally distinct antimicrobial agents, such as rhodamine, chloramphenicol, puromycin, tetraphenylphosphonium, and cetyltrimethylammonium bromide [194]. The reserpine inhibition study showed that in cells with reduced accumulation of ethidium bromide by *bmr*, the reduced drug accumulation was reversed by reserpine and that ethidium bromide efflux from preloaded cells containing *bmr* was completely abolished by reserpine [193]. Random mutagenesis of the *bmr *gene, selection of mutants that lost reserpine sensitivity and DNA sequencing of the *bmr* genes of the mutants, showed that the residues Phe-143, Val-286, and Phe-306 had been replaced, indicating that reserpine interacts with *bmr* at these residues to inhibit drug transport [195, 196]. Another MFS multidrug efflux pump, NorA from *S. aureus*, is closely related to *bmr* [194]. Reserpine also effectively inhibited the ethidium bromide transport activities of NorA [162]. Reserpine also affected the transport activities of two distinct MFS-associated chloramphenicol efflux pumps, CmlR1 and CmlR2, from the Gram-positive bacterium *Streptomyces coelicolor *[119]. The Gram-positive bacterial pathogen *Listeria monocytogenes* harbors the drug efflux pump, Lde, which confers resistance to the fluoroquinolones ciprofloxacin and norfloxacin and is inhibited by reserpine [104]. In our hands, reserpine reduced the MICs of kanamycin and fusidic acid but not of linezolid and lincomycin in cells harboring the multidrug efflux pump LmrS from a methicillin-resistant *S. aureus* clinical isolate [62] suggesting that reserpine does not completely overlap with the substrate binding sites of multidrug efflux pumps and that inhibition may be dependent on the type and nature of the substrate. Unfortunately, reserpine is neurotoxic and is thus not a suitable agent for chemotherapy against infections caused by bacteria harboring MFS multidrug efflux pumps such as NorA [197].

### 8.3. Piperine and MFS MDR Pumps

An alkaloid compound, piperine, from pepper plants, was implicated to be an effective inhibitor of ciprofloxacin efflux pump activity in the Gram-positive bacterium *S. aureus* [198]. Piperine is known to inhibit the activities of a variety of bacterial drug transporters [198–201]. MdeA is known to transport ethidium bromide and Hoechst 33342 across the membrane and to confer resistance to the compounds benzalkonium chloride, doxorubicin, daunorubicin, novobiocin, tetraphenylphosphonium chloride, rhodamine 6G, and virginiamycin [108, 202]. In another study published at about the same time, piperine was found to inhibit drug transport of the multidrug efflux pump, MdeA, from *S. aureus* [199]. When combined with the antibiotic mupirocin, piperine reduced the MIC of the antibiotic against *S. aureus* by several-fold [199]. Piperine was subsequently found to inhibit ethidium bromide efflux activity from the acid-fast bacterium *Mycobacterium smegmatis,* although the affected pump was not definitively identified in that study [203]. The mechanism of modulation for piperine, however, is believed to be direct inhibition of drug efflux.

### 8.4. Inhibitors of NorA Drug Efflux

Two plant-derived alkaloid compounds, called berberine and palmatine, were found to modulate the transporter activity of NorA from *S. aureus* by directly binding to the pump and inhibiting drug transport [179, 204]. Additionally, a proton pump inhibitor agent used to treat gastroesophageal reflux disease omeprazole and newly synthesized derivative analogues of this compound were found to inhibit norfloxacin transport by the multidrug efflux pump NorA [205]. Interestingly, paroxetine, a serotonin reuptake inhibitor, inhibits drug efflux by NorA as well as other non-MFS drug efflux pumps, such as those of the MATE family [69, 206]. Derivatives of the quinolone antimicrobial agent ciprofloxacin, called quinolone esters, were found to be poor substrates for NorA and effective inhibitors of drug efflux by NorA [207]. Recently, derivatives of the COX-2 inhibitor celecoxib were found to be potent inhibitors of NorA [208]. Along these lines, derivatives of 2-phenylquinoline were shown to be good inhibitors of ethidium transport by NorA [209].

### 8.5. Tigecycline and TetA Efflux Pumps

A synthetic derivative of an older antimicrobial agent that successfully restored the efficacy of therapy of bacterial infectious disease treatment was that of tigecycline [210–214]. Originally referred to as a glycylcycline because of a synthetic addition of a glycine moiety to the tetracycline derivative minocycline, tigecycline became an important member of the glycylcycline antimicrobial agents [215, 216]. As such tigecycline was quite effective in treating bacterial infections caused by both Gram-positive and Gram-negative pathogens [217, 218]. Tigecycline was found to circumvent the activity of the class B tetracycline efflux pump (TetB) thus inhibiting the growth of TetB-harboring host bacteria that were resistant to tetracycline [213]. This property of tigecycline is known as a bypass mechanism when considered in light of its relationship to bacterial multidrug efflux. Unfortunately, bacterial resistance to tigecycline has emerged, thus confounding the clinical efficacy of this agent [219]. A multidrug efflux pump belonging to the RND family of transporters was found to be largely responsible for resistance to tigecycline [220]. A tetracycline derivative called DMG-DMDOT (9-(*N,N-*dimethylglycylamido)-6-demethyl-6-deoxytetracycline) is a glycylcycline that was found to be a neither a substrate nor an inhibitor of TetB but rather a good inducer of TetB protein expression by its binding to the TetR repressor protein [221]. Further work will be necessary to enhance the effectiveness of these modulators that bypass multidrug efflux pumps as a mechanism.

### 8.6. Capsaicin and NorA

In a more recently published study, capsaicin, a plant compound used in foods, was found to inhibit the transport of the fluorescent reagent ethidium bromide across the membrane in *S. aureus* cells containing NorA [186]. In the same study, the authors found that capsaicin also prevented *S. aureus *invasion of macrophage cells in culture [186]. Newer and related plant-derived compounds may also be promising toward efflux pump transport modulation.

## 9. Concluding Remarks

In summary, modulation of bacterial drug and multidrug efflux pumps is an important approach to understanding how bacterial resistances may be circumvented in order to restore the clinical efficacy of chemotherapy against presently recalcitrant infectious diseases. We predict that this restorative goal for currently compromised therapeutics will be accomplished by conducting mechanistic molecular studies of drug and multidrug translocation across the membrane and the modulation of both the expression and transport activities of bacterial multidrug efflux pumps.

## Figures and Tables

**Table 1:** Efflux pumps of the MFS family with clinically relevant antibiotics as their substrates.

Bacterium	Efflux pump	Antibiotic substrates	References
*Acinetobacter baumannii *	SmvA	EM	[86, 87]
	CraA	CM	[88]
	CmlA	CM	[89]
*Bacillus subtilis *	Bmr3	FQ, PU	[90]
	LmrB	DR, FQ, LC, PU	[91]
	MdtP	AT, FU, NO, SM	[92]
*Bordetella bronchiseptica *	CmlB1	CM	[93]
*Clostridium difficile *	Cme	EM	[94]
*Clostridium saccharolyticum *	Tet(40)	TC	[95]
*Enterobacter aerogenes *	QepA	FQ	[96]
*Enterococcus faecium *	EfmA	FQ	[97]
*Escherichia coli *	Mef(B)	MC	[98]
	QepA2	FQ	[99]
	EmrAB-TolC	FQ, TE	[100]
	Fsr	TM	[101]
	MdfA	FQ, MC, TE, CM	[102]
*Enterobacter aerogenes *	CmlB	CM	[103]
*Listeria monocytogenes *	Lde	FQ	[104]
*Mycobacterium smegmatis *	LfrA	FQ	[105]
*Salmonella* Typhimurium	EmrAB	NA, NO	[106]
	MdfA	CM, DR, NF, TC	[106]
*Serratia marcescens *	SmfY	NF	[107]
*Staphylococcus aureus *	MdeA	FU, MU, NO, VM	[108]
	NorA	FQ, CM	[109]
	NorB, NorC	FQ	[110, 111]
	MsrA	MC	[108, 112]
	LmrS	LZ, EM, CM, TM, FU	[62]
	SdrM	NF	[113]
	Tet(38)	TC	[111]
*Stenotrophomonas maltophilia *	Smlt0032	MC	[114]
*Streptococcus agalactiae *	MefB	MC	[115]
*Streptococcus pyogenes *	MefA	TC	[116]
*Streptococcus pneumoniae *	MefA, MefE	MC	[117, 118]
*Streptomyces coelicolor *	CmlR1 CmlR2	CM FP	[119]
*Vibrio cholerae *	VceCAB	NA, CM, EM	[120]
	EmrD-3	LZ, EM	[61]

CM: chloramphenicol; DR: doxorubicin; EM: erythromycin; FP: florfenicol FQ: fluoroquinolones; FU: fusidic acid; LC: lincosamides; LZ: linezolid; MC: macrolides; MU: mupirocin; NF: norfloxacin; NO: novobiocin; PU: puromycin; SM: streptomycin; TC: tetracyclines; TM: trimethoprim; VM: virginiamycin.
